# Word Recognition and Frequency Selectivity in Cochlear Implant Simulation: Effect of Channel Interaction

**DOI:** 10.3390/jcm10040679

**Published:** 2021-02-10

**Authors:** Pierre-Antoine Cucis, Christian Berger-Vachon, Hung Thaï-Van, Ruben Hermann, Stéphane Gallego, Eric Truy

**Affiliations:** 1 Integrative, Multisensory, Perception, Action and Cognition Team (IMPACT), Lyon Neuroscience Research Center, CRNL Inserm U1028, CNRS UMR5292, 69675 Bron, France; ruben.hermann@chu-lyon.fr (R.H.); eric.truy@chu-lyon.fr (E.T.); 2 Claude Bernard Lyon 1 University, 69100 Villeurbanne, France; christian.berger-vachon@univ-lyon1.fr (C.B.-V.); hthaivan@gmail.com (H.T.-V.); stephane.gallego@univ-lyon1.fr (S.G.); 3 ENT and Cervico-Facial Surgery Department, Edouard Herriot Hospital, Hospices Civils de Lyon, 69003 Lyon, France; 4 Brain Dynamics and Cognition Team (DYCOG), Lyon Neuroscience Research Center, CRNL Inserm U1028, CNRS UMR5292, 69675 Bron, France; 5 Biomechanics and Impact Mechanics Laboratory (LBMC), French Institute of Science and Technology for Transport, Development and Networks (IFSTTAR), Gustave Eiffel University, 69675 Bron, France; 6 Paris Hearing Institute, Institut Pasteur, Inserm U1120, 75015 Paris, France; 7 Department of Audiology and Otoneurological Evaluation, Edouard Herriot Hospital, Hospices Civils de Lyon, 69003 Lyon, France; 8 Neuronal Dynamics and Audition Team (DNA), Laboratory of Cognitive Neuroscience (LNSC), CNRS UMR 7291, Aix-Marseille University, CEDEX 3, 13331 Marseille, France

**Keywords:** vocoder simulation, normal-hearing, spread of excitation, cochlear implant, speech in noise

## Abstract

In cochlear implants (CI), spread of neural excitation may produce channel interaction. Channel interaction disturbs the spectral resolution and, among other factors, seems to impair speech recognition, especially in noise. In this study, two tests were performed with 20 adult normal-hearing (NH) subjects under different vocoded simulations. First, there was a measurement of word recognition in noise while varying the number of selected channels (4, 8, 12 or 16 maxima out of 20) and the degree of simulated channel interaction (“Low”, “Medium” and “High”). Then, there was an evaluation of spectral resolution function of the degree of simulated channel interaction, reflected by the sharpness (Q10dB) of psychophysical tuning curves (PTCs). The results showed a significant effect of the simulated channel interaction on word recognition but did not find an effect of the number of selected channels. The intelligibility decreased significantly for the highest degree of channel interaction. Similarly, the highest simulated channel interaction impaired significantly the Q10dB. Additionally, a strong intra-individual correlation between frequency selectivity and word recognition in noise was observed. Lastly, the individual changes in frequency selectivity were positively correlated with the changes in word recognition when the degree of interaction went from “Low” to “High”. To conclude, the degradation seen for the highest degree of channel interaction suggests a threshold effect on frequency selectivity and word recognition. The correlation between frequency selectivity and intelligibility in noise supports the hypothesis that PTCs Q10dB can account for word recognition in certain conditions. Moreover, the individual variations of performances observed among subjects suggest that channel interaction does not have the same effect on each individual. Finally, these results highlight the importance of taking into account subjects’ individuality and to evaluate channel interaction through the speech processor.

## 1. Introduction

Modern cochlear implants (CIs) provide unique results in the rehabilitation of severe and profound deafness [1]. Electrode arrays are currently composed of 12 to 22 electrodes depending on the manufacturer [2]. Thanks to multi-electrode technology, speech perception, and quality of life of CI users have been considerably enhanced [3,4]. Nevertheless, an inherent outcome of multiplying the number of channels or electrodes is that it may lead to channel interaction. Indeed, the overlap of electrical fields stimulates a large number of nerve fibers and can create an overlap among the “neural channels”. Depending on the overlap degree, signals, information, neural integration, and neural processing can be degraded [5,6].

A broader spread of excitation increases channel interaction; therefore, it induces a spectral degradation. It is one of the factors leading to a poor spectral resolution, which impairs speech perception, especially in noise. As a consequence, some CI users can’t benefit from the full electrode array [7,8,9,10]. If channel interaction was detected/quantified in one key area or along the electrode array, it could help to establish new fitting processes and refine countermeasures like current focusing, channel deactivation, or channel picking [11,12,13]. However, systematic clinical evaluation of channel interaction seems to be very rare and therefore it is not objectively taken into account in the fitting process of CIs.

Channel interaction caused by a broad spread of excitation can be evaluated by psychophysical or electrophysiological techniques [14,15,16]. Psychophysical tuning curves (PTCs), have been largely used to quantify frequency selectivity, or channel interaction, in CI users [17,18].

In general, PTCs are considered to be time-consuming when they are measured with traditional forced-choice adaptative methods. To reduce the testing-time, a fast method has been developed by Sek et al. (2005, 2011) based on the Bekesy-tracking procedure and evaluated with normal-hearing (NH) and hearing-impaired subjects [19,20]. Kreft et al. (2019) adapted this procedure to CI users and reported that it was 3-times faster (around 20 min versus 60 min) but its repeatability was lower than the traditional forced-choice method [21]. Additionally, some authors suggested that other methods, like froward-masking electric compound action potentials (ECAP) or spectral ripple discrimination, for example, were quicker than PTCs but without comparing testing-times [22,23].

In recent experiments, psychophysical methods such as forward masking PTCs did not always appear to be strong predictors of speech perception outcomes in CI users [18,24]. Although, some studies reported encouraging correlations with speech perception scores. For example, Anderson et al. (2011) described a positive correlation between the inverse of the PTC bandwidth and sentence recognition. Additionally, Boëx et al. (2003) found a negative correlation between the level of forward-masking between the different intracochlear electrodes and the consonant identification performance [22,25].

Nevertheless, some authors underlined the fact that the PTCs are, in general, measured by direct electrical stimulations and do not take into account the constraints introduced by the speech processor, and may not reflect the frequency selectivity in usual conditions. This could hinder the comparison with speech recognition [17,18,26].

An effective way to study speech-processor constraints is to use a vocoder simulation [27]. Dorman et al. (2000) stated that experiments conducted with such a simulator are reliable and comparable with experiments conducted with CI users [28]. Vocoder simulations are well correlated with the best performing CI users [13] and enable the assessment of speech recognition scores under various and controlled conditions (spread of excitation for example). Like a cochlear implant, the noise vocoder degrades temporal fine structures. Because the frequency channels are created with random-phase narrow-band noises, the output of the vocoder does not restore the temporal fine structure of the original signal [29,30]. Additionally, simulations could also evaluate tuning curves sharpness under vocoded sounds in NH subjects [31]. Finally, the use of a vocoder simulation with NH subjects allows the use of repeated measures designs where the subjects are their own controls. In absence of confounding factors, it enables an efficient management of the tested factors and it leads to powerful statistical analyses.

This study aims at determining if word recognition in noise is correlated with frequency selectivity when channel interaction is simulated with a cochlear implant simulator on NH subjects. To do so, we used a 20-channel noise-band vocoder to mimic the signal processing of a common CI and we added an algorithm that simulated spread of excitation. We measured the word recognition in noise as a function of the number of maxima and the level of simulated spread of excitation. We also assessed the spatial tuning curves sharpness function of the spread of excitation and we evaluated the statistical association between the word recognition in noise and the spatial tuning curves sharpness.

## 2. Materials and Methods

### 2.1. Subjects

A total of twenty native French speakers (10 females, 10 males) aged from 19 to 40 years old (mean = 26.3 years, SD = 6.4 years) took part in the study. They received a financial compensation for their participation. Pure tone audiometry was performed on all participants to verify that their hearing was normal (average hearing loss below 20 dB HL on each ear for 500, 1000, 2000, and 4000 Hz) following the recommendations of the International Bureau for Audiophonology [32]. Subjects reported no significant history of audiological or otological problems such as ear surgery or repeated otitis media during childhood. Moreover, they had no self-report history of neurological impairments or psychiatric disorders.

Written informed consent was obtained from the subjects before their inclusion. The study was conducted following the guidelines of the French Good Clinical Practice, the Helsinki Declaration in its latest version, and the recommendations of the ICH (International Council on Health). The ethics committee CPP East-IV issued a favorable opinion on the realization of this study.

### 2.2. Hardware

Testing took place in a double-wall sound-treated room. All stimuli were generated by a standard PC connected to an external sound-card M-Track MkII (M-Audio, Cumberland, RI, USA) and were presented to the subjects using TDH39 headphones (Telephonics Corporation, Farmingdale, NY, USA). Sound levels were controlled by a MADSEN Orbiter922 clinical audiometer (GN Otometrics A/S, Traastrup, Danemark). The audiometer is used routinely in clinical practice and is calibrated yearly.

### 2.3. Vocoder Signal Processing

**Figure 1:**
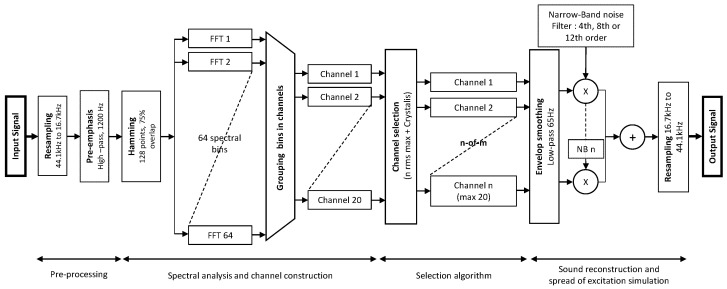
Block diagram of the CI simulation/vocoder.

All signal processing was implemented in MATLAB (MathWorks, Natick, MA, USA). A 20-channel noise-band vocoder was used to mimic the signal processing of a Saphyr ^®^ SP sound processor (Oticon Medical, Vallauris, France). A Crystalis fitting strategy was simulated, which is a sound coding strategy commonly used in Oticon Medical devices (Oticon Medical, Vallauris, France) (Figure 1).

First, the audio signals (recorded at 44.1 kHz) were down-sampled to 16.7 kHz. A high-pass pre-emphasis filter (Infinite Response) was applied to the input signal (fc = 1200 Hz). The signal was windowed by a 128-sample Hamming window (~7.7 ms) with a temporal-overlap of 75% resulting in an inter-frame interval of approximately 1.9 ms.

Fast Fourier Transform (FFT) was computed on each windowed part of the signal, resulting in a 64-bin spectrum. The first two and the last two bins were rejected; the remaining 60 bins (FFT coefficients) represented the frequencies between 195 and 8008 Hz (130.2 Hz step). Then, they were distributed into 20 non-overlapping analysis channels (Table 1).

For each channel, a root-mean-square (RMS) was computed by using the FFT components representing the frequencies within the respective channels. In each temporal frame, the Crystalis (n-of-m) selection rules were applied: only the “n” channels with the highest RMS amplitude were kept (the others were set to zero). The remaining “n” channels were then compared to the highest RMS and the channels with an RMS-amplitude lower than RMSmax minus 45 dB were set to zero.

After channel selection, temporal envelopes were reconstructed by modulating Hamming windows with the RMS-amplitudes and by using an “overlap-and-add” procedure (75% overlap). A second-order Butterworth filter with a 65-Hz cutoff frequency (half the gap between the frequency bins) was used to smooth the envelopes [33,34,35]. Then, the temporal envelopes were used to modulate narrowband noises with the same cutoff frequencies as the corresponding analysis channels (Table 1). The narrowband noises were obtained by filtering a broadband Gaussian noise according to the analysis frequency bands. At this stage, 4th, 8th, and 12th order Butterworth filters (respectively, −24, −48 and −72 dB/oct slope) were used to simulate “High”, “Medium” and “Low” spread of excitation.

The resulting modulated narrowband noises were summed and the output signal energy was leveled to the input signal energy and if necessary, normalized between −1 and 1 to avoid peak-clipping. When the process was complete, the signal was resampled to 44.1 kHz and stored in a “wav” file.

### 2.4. Speech Audiometry in Noise

The speech material was French dissyllabic words uttered by a male speaker. The words were extracted from Fournier’s lists [36]. Speech and noise were summed at the required signal to noise ratio (SNR) before being processed by the vocoder. The noise used here was a cocktail-party noise (a mixture of chatter and tableware noises).

Sounds (words + noise) were presented to the subjects’ right ear using headphones connected to a clinical audiometer that calibrated the sound level at 65 dB SPL. Subjects were instructed to repeat each word after it was presented to them. A word-list incorporates 10 dissyllabic words and the error unit was the syllable that led to final scores between 0 and 20, then converted to percentage.

There was a short training session before the actual test to accustom the subject to the vocoded sounds and to be sure that he or she understood the instructions. Training words were extracted from the first list of Lafon’s dissyllabic words [37] and they were presented to the subject after being processed by the vocoder with the following parameters: +18 dB SNR, 16 maxima (16 out of 20), and “Low” spread (72 dB/octave filter slope). This training session was not part of the experiment.

For the actual testing session, a combination of three conditions was attributed randomly to a Fournier’s word list:

SNR: −3, 3, and 9 dB (mixed before vocoding, as it is the case with CIs)

Number of maxima: 4, 8, 12, and 16 (out of 20)

Spread of excitation: “Low” (−72 dB/octave filter slope), “Medium” (−48 dB/oct) and “High” (−24 dB/oct).

The combination led to 36 different conditions (so 36 lists were presented to each subject).

### 2.5. Psychophysical Tuning Curves

#### 2.5.1. Stimuli

We chose the stimuli to be able to reproduce this experiment with CI users with a Digisonic SP cochlear implant. We determined the frequencies for which only one electrode was activated. (see Table 2).

Pure tones that activated only one electrode were recorded by sweeping the frequencies from 190 up to 8000 Hz with a 1 Hz step. Sine-waves were sent to a Saphyr^®^ SP sound processor via an auxiliary cable and we recorded the activated electrodes using a Digispy interface provided by Oticon Medical. Three-second sine-waves were generated by a MATLAB script and the PC sound card was set on 100% volume. Levels were adjusted at 50% of the stimulation dynamic using the volume wheel on the auxiliary cable. The sound processor settings are indicated in Table 3.

Then, to establish the PTCs, the sounds were presented to the right ear. A MATLAB script generated the stimuli and the sound levels were adapted according to the answers given by the subject (see Section 2.5.2).

The probe was set to fp = 2226 Hz which is the center “frequencies for single-channel activation” of the 8th channel. Maskers matched with channels 11 to 5, fm = 1440.5, 1637, 1898.5, 2226, 2619, 3143 and 3798 Hz.

The 110-ms masker was followed by the 20-ms probe with no delay. Both stimuli were gated with 4-ms raised-cosine-squared ramps before entering the vocoder and gated again after signal processing to ensure no temporal artifacts.

Three tuning curves were established for each subject (one for each level of spread of excitation: “Low”, “Medium” and “High” as described above). Stimuli were obtained by presenting pure-tones at the input of the vocoder. This is equivalent to measuring PTCs with narrowband noises that have different slopes.

#### 2.5.2. Procedure

A three-interval-forced-choice (3IFC) [38], two-up one-down forward masking paradigm was used to determine the masked thresholds. The volume of the masker was increased when the subject correctly identified the position of the “masker–probe” sequence twice in a row. The volume of the masker was decreased after one wrong answer. Each PTC took approximately one hour to complete. A break was proposed between each session.

For each listener, a hearing threshold and a maximum acceptable level were measured for the maskers and the probe. This was repeated before each PTC run as the stimuli changed with the simulated spread of excitation.

A short training period was performed before the actual test to be sure that the subject understood the instructions and could hear and identify the probe frequency at the beginning of each run.

A total of three sounds were presented to the subject, one contained the masker–probe sequence, the two others contained the masker only. The goal was to identify the position of the masker–probe sequence among the three intervals (1st, 2nd, or 3rd position) and to enter the answer on a numeric keypad from a remote keyboard in front of the subject by pressing 1, 2, or 3. There were no visual cues.

The level of the probe frequency was fixed at 20% of the dynamic range. Starting at a level of 10 dB SL, the masker sound level was adaptively changed with a 4 dB step for the first three reversals, decreased to 2 dB for reversals three to six, and 1 dB for the last six. There were 12 reversals inside a run and the masked threshold was defined in dB SPL as the average masker level at the last six reversals.

### 2.6. Tuning Curves Fitting and Q10dB

Each PTC was fitted with two quadratic functions, one on the low-side and one on the high-side around the probe frequency (R^2^: Mean = 0.980; SD = 0.037; min = 0.778; max = 1.00). Slopes on both sides were considered monotonic, so if a masked threshold did not follow this rule with a deviation higher than 10 dB, it was not taken into account for the regression. Following this rule, the typical fitted-function included all the seven masking thresholds except for three subjects: subject S04 (6 points for the “Low” spread curve and 5 points for the “Medium” curve), S07 (6 points, “Medium” spread) and S19 (6 points, “Low” spread). Moreover, S16 did not manage to perform the test and the PTCs could not be established so, the results were analyzed for 19 subjects (out of 20). From the fitted PTCs, we then characterized channel interaction using the Q10dB as a sharpness factor. Q10dB was calculated by dividing the probe frequency by the PTC bandwidth (BW) at 10 dB above the tip level (Q10dB = 2226/BW10dB).

### 2.7. Statistical Analyses

Statistical analyses were performed using Addinsoft XLSTAT 2020 (Addinsoft Inc., New York, NY, USA) and RStudio Version 1.1.456© 2021–2018 (RStudio Inc., Boston, MA, USA).

Before analysis, word recognition scores were transformed into rationalized arcsine units (RAUs) with a correction for the small number of items [39]. Converting word recognition proportion scores to RAUs allows more appropriate statistical analyses and attempts to minimize floor and ceiling effects [40].

Word recognition scores were evaluated by a repeated-measures ANOVA using linear mixed models with three factors of interest: SNR (9 dB, 3 dB, and −3 dB SNR), number of maxima (4, 8, 12, and 16 out of 20), and level of spread of excitation (“Low”: −72 dB/octave, “Medium”: −48 dB/octave, “High”: −24 dB/octave), and finally subject as the random factor. Then, 2-by-2 comparisons were made with bilateral paired-samples *t*-tests, and significance levels were adjusted according to the Bonferroni correction.

For Q10dB, a repeated-measures ANOVA, using linear mixed models, was performed to determine if there were significant differences in Q10dB between the levels of spread of excitation. Then, 2-by-2 comparisons were made with paired-samples *t*-tests. Significance levels were adjusted according to the Bonferroni correction.

A linear correlation was measured between mean intelligibility scores (in RAUs, calculated across the SNRs) and Q10dB (3 points per subjects, one for each level of spread of excitation). To account for repeated measurements within the same subjects, we performed repeated-measures correlations using the rmcorr package in R. Indeed, this technique takes into account the non-independence between the measurements and uses an analysis of covariance to consider inter-individual variability. Therefore, rmcorr calculates parallel regression lines (same slopes, varying intercepts) to fit each participant in the best possible way [41].

Finally, for each subject, we compared the evolution of the average word recognition in noise function of the evolution of Q10dB by calculating the difference between the scores for the “Low” and the “High” spread of excitation. A Spearman correlation was performed between those variables.

## 3. Results

### 3.1. Speech Audiometry in Noise

**Figure 2:**
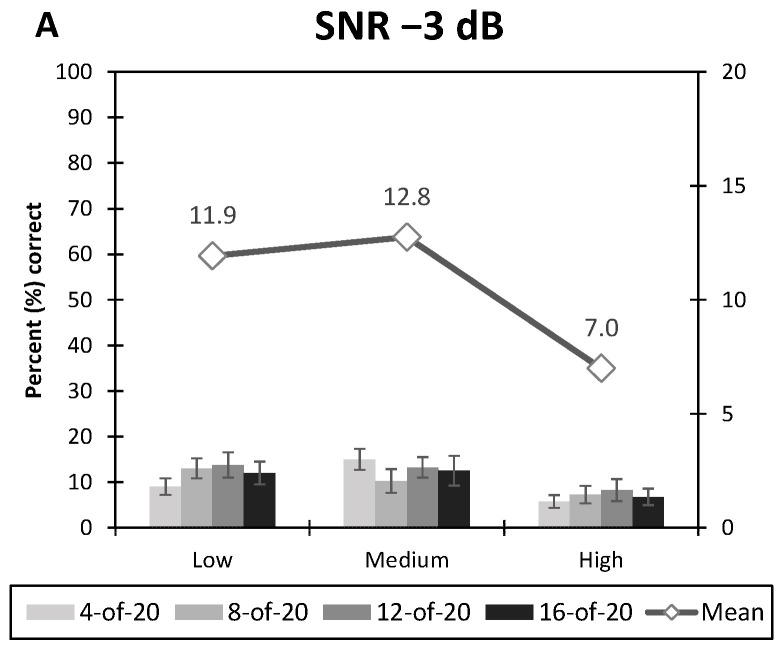

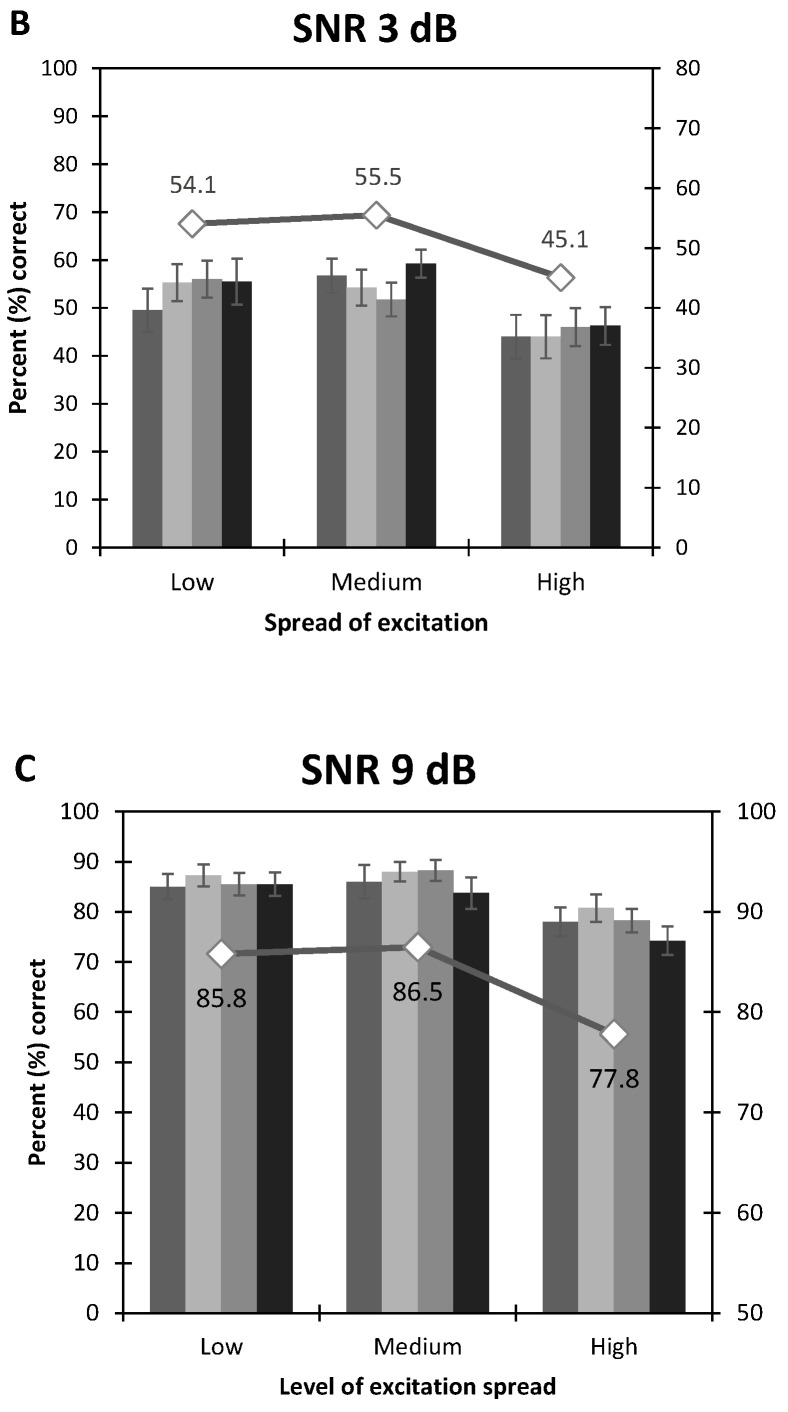
Average syllable recognition scores (in percent correct) function of the spread of excitation and number of maxima. Each number of maxima is represented by a different grey bar, and average performances for a single level of spread of excitation (across the number of maxima) are represented by a grey line with diamonds. The left scale is for the bars and scale on the right, is for the averages across maxima. (**A**) Condition −3 dB of signal-to-noise ratio, (**B**) Condition 3 dB SNR, and (**C**) Condition 9 dB SNR. Error bars represent ±1 standard error of the mean.

Word recognition scores are displayed as percentages for more clarity. However, statistical analyses were performed on RAUs scores as described above. Scores in RAUs ranged from −12.78 to 112.78 RAUs, corresponding to recognition scores of 0% to 100%, respectively. Figure 2 shows an overview of the data. The results are split into three graphs, one for each SNR, and organized to ease visualization and interpretation.

The repeated measure ANOVA (mixed models) revealed a significant main effect of:

- The Spread of excitation: F2, 677 = 23.80, *p* < 0.0001,
- The SNR: F2, 677 = 999.32, *p* < 0.0001,
- No effect of the Number of Maxima: F3, 677 = 0.60, *p* = 0.61.

Additionally, the two-way interactions between the factors were not significant:

- Spread of excitation × Number of Maxima: F6, 677 = 0.75, *p* = 0.61,
- Spread of excitation × SNR: F6, 677 = 0.18, *p* = 0.95,
- Number of Maxima × SNR: F6, 677 = 0.75, *p* = 0.61.

**Figure 3:**
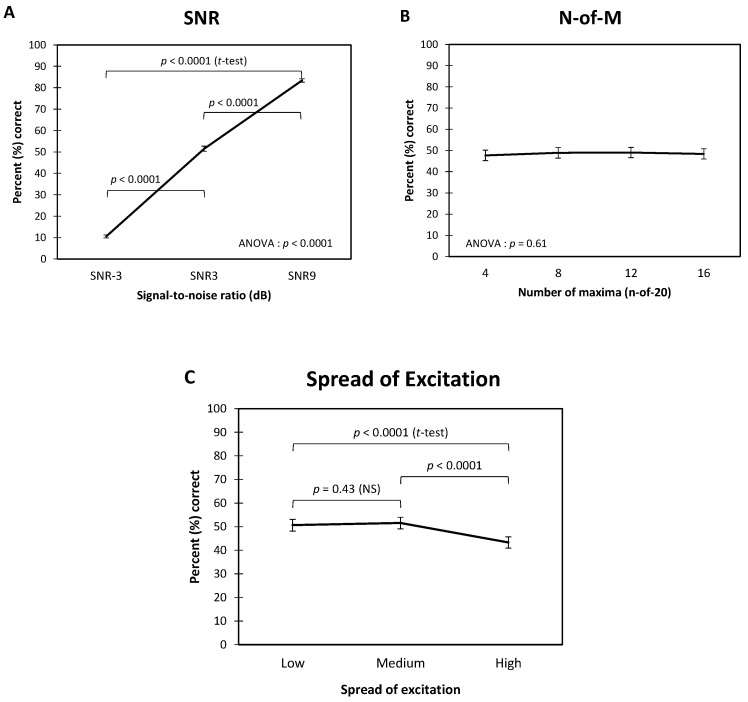
Results of the 3-way repeated measures ANOVA and results of the 2-by-2 comparisons (Student’s *t*-tests) made on scores transformed in rationalized units (RAU). (**A**) Average syllable recognition scores (in percent correct) across the signal-to-noise ratios. (**B**) Average across the number of maxima. (**C**) Average across the levels of spread of excitation. Error bars represent ±1 standard error of the mean.

Average recognition scores for each factor (in percent correct) are presented in Figure 3 and Table 4. We can see that the average scores across the number of maxima remained around 50%. Average scores across SNRs ranged from around 11% at −3 dB SNR to 83% at 9 dB SNR. Furthermore, all three 2-by-2 comparisons were significant (*t*-tests, *p* < 0.0001). Then, the 2-by-2 comparisons revealed a significant decrease in the average scores for the “High” level of spread of excitation compared to the “Low” and “Medium” levels (from around 50% to 43%). T-tests revealed that the average score for a “High” level of spread of excitation was different from the two others (“Low” vs. “High”: *p* < 0.0001; “Medium” vs. “High”: *p* < 0.0001; “Low” vs. “Medium”: *p* = 0.43).

### 3.2. Psychophysical Tuning Curves

**Figure 4:**
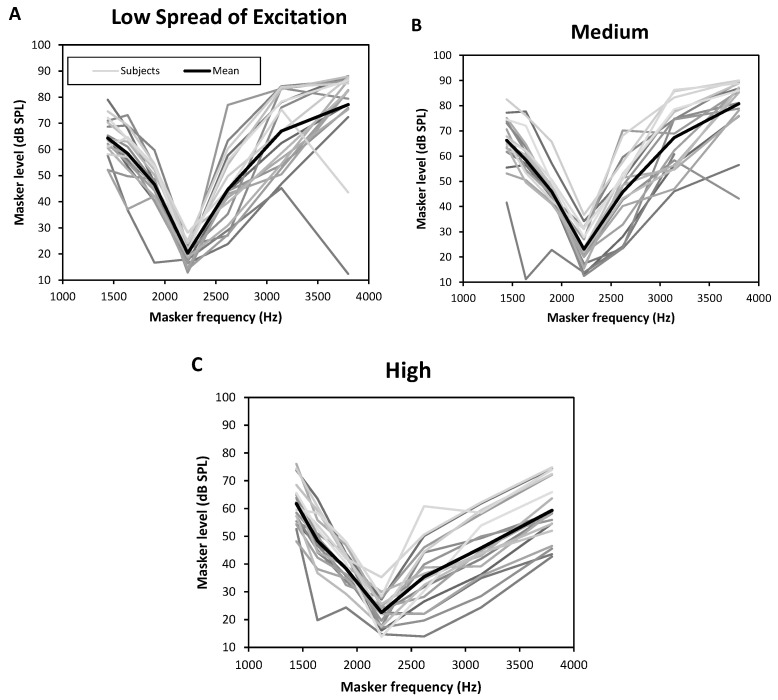
Measured psychophysical tuning curves (PTC). Masking thresholds (in dB SPL) function of the masker frequency (fm = 1440.5, 1637, 1898.5, 2226, 2619, 3143 and 3798 Hz). Each subject is represented by a different grey curve and the average curve is in black with white dots. (**A**) PTCs for “Low” spread of excitation. (**B**) PTCs for “Medium” spread. (**C**) PTCs for “High” spread.

Individual and average PTCs are displayed in Figure 4 (masking threshold in dB SPL function of masker frequency in Hz). Table 5 gives an overview of the results. First, looking at the shape of the PTCs, we can see that there is a noticeable heterogeneity between subjects. Furthermore, this heterogeneity seems to be larger on the high-frequency side of the curves, with standard deviations of approximately 18, 12, and 10 dB SPL versus approximately 7, 9, and 8 dB SPL on the low side. Finally, the “High” level of spread of excitation seems to flatten the curve while the “Low” and “Medium” levels give very similar shapes.

**Figure 5:**
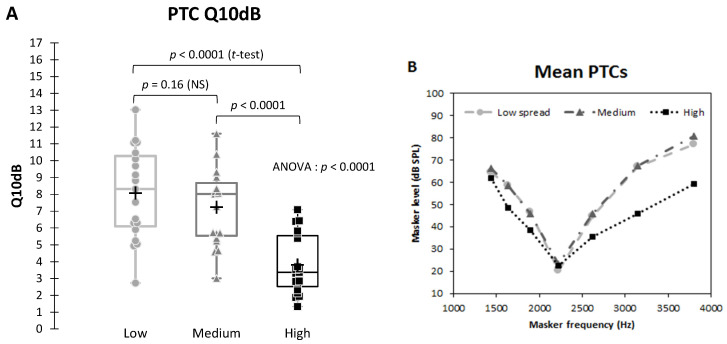
Comparison of the sharpness (Q10dB) of the average psychophysical tuning curves (PTC) function of the level of spread of excitation. Results of the repeated measure ANOVA and results of the 2-by-2 comparisons (Student’s *t*-tests). (**A**) Boxplots showing Q10dB: the horizontal line within the box indicates the median; means are indicated by a plus sign; edges are the 25th and 75th percentiles, whiskers the most extreme data points. Each dot represents one subject. (**B**) Average tuning curves for the three levels of spread of excitation.

These changes in shape had an impact on the Q10dB values as we can see in Figure 5. The average Q10dB for the “Low” spread was approximately 8, for the “Medium” level around 7, and a noticeable decrease for the “High” level with a Q10dB of 3.

The repeated measure ANOVA (mixed models) revealed a significant main effect of the level of spread of excitation (F2, 36 = 38.49, *p* < 0.0001). The 2-by-2 tests showed a significant difference between the Q10dB at “High” level of spread of excitation and the two others (“Low” vs. “High”: *p* < 0.0001; “Medium” vs. “High”: *p* < 0.0001; “Low” vs. “Medium”: *p* = 0.16).

### 3.3. Correlation between Word Recognition and PTC Sharpness

**Figure 6:**
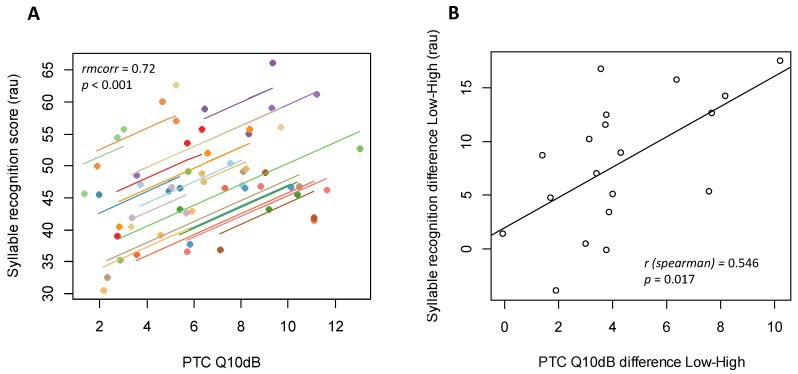
Average syllable recognition scores (in rau) function of Q10dB (**A**) Repeated measures correlation (rmcorr). Measures from the same participant are given the same color, with corresponding lines to show the rmcorr. There are 3 dots per participants, one for each level of spread of excitation. (**B**) Average syllable recognition difference function of Q10dB difference between “Low” and “High” spread of excitation simulation. Each dot represents one subject.

We assessed the relationship between the PTC sharpness (Q10dB) and the word recognition (in RAUs) (Figure 6). First, repeated measures correlations indicated a strong positive association (*rmcorr* = 0.72, *p* < 0.001). Then, we compared the evolution of the performances between the “Low” and “High” level of simulated spread of excitation: the difference of word recognition and the difference of Q10dB were positively correlated (average values measured across all the SNRs) (*r_spearman_* = 0.55, *p* = 0.017).

## 4. Discussion

In this study, we investigated the impact of simulated channel interaction on the word recognition in noise and on the frequency selectivity of 20 NH subjects. We used a 20-channel vocoder with a simulation of spread of excitation.

Word recognition in cocktail-party noise was evaluated on disyllabic words by varying: the SNR (−3, 3 and 9 dB SNR in front of the vocoder); the number of selected maxima (4, 8, 12, and 16 out of 20) and the spread of excitation (synthesis filter slopes of −24, −48 and −72 dB/octave). Frequency selectivity was characterized by the Q10dB of forward-masked PTCs, measured with sounds processed by the vocoder and the simulation of the spread of excitation.

This experiment is a simulation and should be completed by results from a transposed experiment with CI users. The NH subjects where relatively young and some age categories are underrepresented (between 30 and 50 years old), which could influence de results. The choice of the Fournier’s dissyllabic words appeared to be adequate. Nevertheless, it should be noted that the recognition of Fournier’s words can be influenced by the lexical knowledge of the subject.

The main finding of this study is that, within individuals, simulated channel interaction, correlates with PTCs selectivity (Q10dB) and correlates with speech recognition in noise.

The motivation to choose a cocktail-party noise for speech audiometry in noise was its complexity and that it recreates conditions of realistic listening environments, such as schools, restaurants, and other social gatherings. Cocktail-party noise is a broadband fluctuating noise and it is very close to the long-term speech spectrum. Therefore, it induces interferences due to amplitude modulation and informational masking which play an important role in CI users’ speech recognition. The similarities between the target and the masker, exacerbated by an impaired spectral resolution, increase attentional resources needed to differentiate the speech signal and the cocktail-party noise [42,43,44].

It was observed a speech recognition plateau with around four channels in quiet [45,46]. Around 10 channels are necessary to reach a plateau under more adverse conditions (e.g., noise, difficult speech material, etc.) [7,27]. Recently, it was observed that intelligibility could continue to improve with a larger number of channels [47,48]. In vocoder studies, even if a plateau was also observed, the listening effort seems to be reduced when more channels are used [49,50]. In our experiment, word recognition in noise was not significantly changed by varying the number of selected maxima. This result may be due to the characteristics of our vocoder. The smoothing performed by the “overlap-and-add” reconstruction and the 65 Hz low pass filtering partially filled the temporal gaps created by the channel picking. These steps do not entirely suppress the effect of channel-picking but they may have an impact on the final results. In these conditions, subjects would already have reached a plateau of performance and, in our experiment, increasing the number of selected channels beyond four would make no difference.

In general, channel-picking vocoders use smoothing filters that follow the analysis rate. For example, Dorman et al. (2002) used a 400 Hz cutoff frequency and found a significant effect of the number of maxima on speech recognition. In quiet, performances reached a plateau for 6-of-20 maxima. In noise, 9-of-20 maxima were needed. In this study, the effect of channel interaction was not investigated. There was a constant overlap between the analysis channels in the experiment [51] (sixth order Butterworth filters, −36 dB/oct). Then, Bingabr et al. (2008) investigated the effect of varying the degree of simulated channel interaction with a fixed-channel vocoder (4, 8 and 16 channels). The data showed a significant interaction between the number of channel and spread of excitation. They concluded that recognition of sentences in noise is likely to be improved by reducing channel interaction and by improving the spectral resolution to 8–16 channels [52]. Therefore, simulated channel interaction may not suppress the effect of changing the amount of spectral information on speech recognition. On the contrary, Verschurr et al. (2009) investigated the effect of 4-of-7 and 12-of-20 strategies with simulated channel interaction and found no substantial changes in consonant recognition performances [13]. They also stated that reduced consonant recognition in better performing cochlear implant users was mainly due to cochlear implant processing and not to channel interaction.

These studies give some clues but it should be noted that varying the total number of channels is not directly comparable to varying the number of maxima with a channel-picking coding strategy. Channel-picking strategies modulate the relative importance of each channel so it has an effect on spectral contrast and spectral resolution, depending on the proportion of selected channels. Several investigations found a significant decrease in speech-in-noise recognition and supported the idea that channel interaction can affect speech perception outcomes [53,54].

As hypothesized, simulated channel interaction significantly decreased word recognition in noise in this study. Notably, word recognition was decreased when the spread of excitation was at the highest level (−24 dB/octave) compared to the two lower situations (−48 and −72 dB/octave). It seems to indicate a threshold effect. This is consistent with some recent studies. For example, In Gaudrain and Baskent (2018), the recognition of a shift in the spectral envelope of a syllable significantly changed at −24 dB/octave compared to −48 and −72 dB/octave filter slopes [55]. In Jahn et al. (2019) vowel and consonant recognition dropped significantly for −15 dB/octave filter slopes compared to −30 and −60 dB/octave conditions [34]. This threshold effect may be due to the smaller difference of slope between −48 and −72 dB/octave filters than between −24 and −48 dB/octave filters.

This confirms that poor spectral resolution due to channel interaction negatively impacts speech perceptions in quiet and in noisy conditions.

In our study, frequency selectivity (reflected by the Q10dB) was significantly decreased by spatial spread and results showed, like for speech perception, a threshold effect. Again, the spread of excitation at the highest level (−24 dB/octave) was different from the two other situations (−48 and −72 dB/octave).

A small number of studies have investigated the effect of simulated spatial spread of excitation on the shape of PTCs. One of them, Langner et al. (2016) showed an improvement of the PTCs Q10dB by using a dynamic compression algorithm to restore frequency selectivity in CI users and NH subjects listening to a vocoder [31]. This kind of simulation seems equivalent to measuring PTCs and varying the frequency range of the maskers.

Concerning the makers’ frequency range, Kluk and Moore (2004) have measured Q10dB with NH subjects using simultaneous masking for 1 and 4 kHz probe frequencies (pure tones). They tested noise-maskers of 80, 160, and 320 Hz wide and found a decrease in Q10dB when increasing the noise-maskers’ bandwidth. They suggested that only a part of the masking noise passed through the auditory filter. Indeed, in their experiment, for the two wider noise-maskers (160 and 320 Hz), the bandwidths were greater than the equivalent rectangular bandwidth (ERB) at the reference frequency [56].

Using wide maskers with different power decay seems to have the same effect. In our case, the probe was a narrow-band noise with a center frequency of 2214 Hz and the ERB for this frequency is approximately 264 Hz. The probe sound and the “high-frequency” maskers were wider than 264 Hz (Table 1). It may explain the broadening of the PTCs on the “high-frequency” side.

Because PTC measures have been associated with small correlations with speech perception of CI users [18,22,26], some methods like spectral ripple discrimination tests have been used [35,57]. In our experiment, the result of the repeated measure correlation showed a strong within-subject correlation between Q10dB and word recognition in noise when changing the simulated spread of excitation (Figure 5). It means that for each subject there is a strong correlation between word recognition and frequency selectivity reflected by the Q10dB parameter. The within subject correlation obtained with a vocoder could also suggest that measuring PTCs through the speech processor is worthwhile to be explored because processors induce constraints not taken into account in experiments measuring PTCs by direct electrical stimulations. Q10dB measured through the speech processor would be closer to channel interaction experienced by each CI users. The same protocol as the one presented here could be transposed to CI users.

Finally, our subjects were considered as NH people but the results showed a variable resilience between individuals. The improvement of word recognition in noise was correlated with the improvement of Q10dB in NH listeners using a vocoder CI simulator (Figure 6). As the simulated channel interaction was the same for all, the effects were heterogeneous between subjects. It seems that people are not equal facing spread of excitation and for CI users it may be the same case. Despite the documented variability of speech in noise performances among people with normal-hearing, this experiment shows that there is also a variable resistance to spectral smearing. In some cases, when spectral smearing was increased the speech recognition and the Q10dB barely changed.

## 5. Conclusions

In this study, when we changed the degree of simulated channel interaction there was a strong within-subject correlation between PTCs frequency selectivity and average speech recognition in noise. This result supports the hypothesis that forward masked PTCs are correlated to individual word recognition in noise. Furthermore, while the same simulation was expected to have mainly the same effect on NH subjects, changing simulated channel interaction did not cause the same outcomes. The results showed a correlation between the evolution of the frequency selectivity and the average speech recognition in noise across the “Low” and the “High” degree of simulated spread of excitation. Then, between the two lightest simulated channel interaction frequency selectivity, word recognition in noise did not significantly change while the strongest interaction impaired significantly the scores. This result shows a threshold effect, the channel interaction has to be sufficiently wide to induce a modification of performances. Additionally, as the vocoder mimics a CI speech processor, these findings highlight the importance of measuring PTC through the speech processor to take into account the signal processing. Finally, it seems useful to investigate with CI users the individual impact of channel interaction on PTCs frequency selectivity and speech recognition in noise. As vocoder simulations are good predictors of CI users’ performances, we could expect a correlation between frequency selectivity and word recognition within individual CI users.

## Figures and Tables

**Table 1:** Centre and cutoff frequencies of the vocoder. Number of bins (FFT coefficients) per channel.

Channel	Lower Cutoff (Hz)	Higher Cutoff (Hz)	Center Frequency (Hz)	Bin(s) Per Channel	Filter Bandwidth (Hz)	Equivalent Rectangular Bandwidth (Hz)
20	195	326	261	1	131	53
19	326	456	391	1	130	67
18	456	586	521	1	130	81
17	586	716	651	1	130	95
16	716	846	781	1	130	109
15	846	977	912	1	131	123
14	977	1107	1042	1	130	137
13	1107	1237	1172	1	130	151
12	1237	1367	1302	1	130	165
11	1367	1497	1432	1	130	179
10	1497	1758	1628	2	261	200
9	1758	2018	1888	2	260	228
8	2018	2409	2214	3	391	264
7	2409	2799	2604	3	390	306
6	2799	3451	3125	5	652	362
5	3451	4102	3777	5	651	432
4	4102	4883	4493	6	781	510
3	4883	5794	5339	7	911	601
2	5794	6836	6315	8	1042	706
1	6836	8008	7422	9	1172	826

**Table 2:** Frequencies for single electrode activation.

Channel	Lowest Activation-Frequency (Hz)	Highest Activation-Frequency (Hz)	Center Frequency (Hz)
20	195	265	230
19	390	396	393
18	521	527	524
17	652	658	655
16	783	789	786
15	914	920	917
14	1045	1051	1048
13	1176	1182	1179
12	1307	1312	1310
11	1438	1443	1441
10	1569	1705	1637
9	1830	1967	1899
8	2092	2360	2226
7	2485	2753	2619
6	2878	3408	3143
5	3533	4063	3798
4	4188	4848	4518
3	4973	5765	5369
2	5890	6813	6352
1	6938	8115	7527

**Table 3:** Sound processor settings while measuring the “activation bandwidths”.

Parameter	Setting
Min. Stim	9 ns
Max. Stim	52 ns
Strategy	Crystalis XDP
Stimulation	500 Hz
Maxima	16
Compression	Linear (personalized)
Dynamic range	26–105 dB SPL
Audio input	Auxiliary only (0 dB Gain)

**Table 4:** Average syllable recognition scores for each factor and variation.

Factor	Variation	Unit	Mean	Standard Deviation
SNR	SNR-3	%	10.6	10.8
		rau	6.5	16.1
	SNR3	%	51.5	18.6
		rau	51.3	17.9
	SNR9	%	83.4	12.4
		rau	84.9	16.2
Number of Maxima	4-of-20	%	47.7	33.4
		rau	47.1	36.7
	8-of-20	%	48.8	33.7
		rau	47.9	36.9
	12-of-20	%	49.0	32.5
		rau	48.1	35.2
	16-of-20	%	48.4	32.6
		rau	47.1	36.1
Spread of excitation	Low	%	50.6	33.3
		rau	50.1	36.2
	Medium	%	51.5	33.0
		rau	51.1	36.4
	High	%	43.3	32.2
		rau	41.4	35.4

**Table 5:** Average masking thresholds (in dB SPL) function of the simulated spread of excitation.

Frequency (Hz)/Masking Threshold (dB SPL)	1441	1637	1898.5	2226	2619	3143	3798
	**Low spread**						
Mean	64.4	58.4	46.5	20.3	44.6	67.0	77.2
Standard error	6.9	9.6	8.8	4.3	13.9	13.9	18.2
Min	52.1	36.8	16.6	12.8	23.8	45.2	12.3
Max	79.0	73.1	59.8	28.2	77.0	84.2	88.1
	**Medium**						
Mean	66.2	58.4	45.9	23.1	45.7	67.3	80.8
Standard error	9.2	13.9	7.9	7.3	13.8	12.6	12.1
Min	41.5	11.2	22.8	12.4	23.4	46.0	43.1
Max	82.5	77.7	65.6	36.7	70.2	86.3	90.0
	**High**						
Mean	61.8	48.4	38.4	22.6	35.4	45.7	59.3
Standard error	7.5	9.9	5.8	5.5	11.8	10.9	10.4
Min	48.2	19.8	24.4	13.8	13.9	24.3	42.7
Max	76.1	63.6	48.1	35.3	60.8	62.2	74.8

## Data Availability

The data presented in this study are available on request from the corresponding author. The data are not publicly available due to privacy protection.
